# Air-Exposure- and Reoxygenation-Stimulated Expressions of Caspase-3 and Induction of Apoptosis in the Central Nervous System of the Crab Erimacrus isenbeckii

**DOI:** 10.3390/cells14110827

**Published:** 2025-06-02

**Authors:** Elena Kotsyuba, Vyacheslav Dyachuk

**Affiliations:** A.V. Zhirmunsky National Scientific Center of Marine Biology, Far Eastern Branch, Russian Academy of Sciences, Vladivostok 690041, Russia; epkotsuba@mail.ru

**Keywords:** dopamine, caspase-3, apoptosis, crustaceans

## Abstract

Air exposure stress during live transport and subsequent reoxygenation are factors in the development of molecular/pathological and compensatory/adaptive responses. They affect the physiological functions and survival of economically important invertebrate species, in particular, crustaceans. In this study, we consider the effects of anoxia and subsequent reoxygenation on the physiological responses, signaling pathways involved in stress, and cell apoptosis in the central nervous system (CNS) of the horsehair crab, *Erimacrus isenbeckii*. The results showed that 1 day of air exposure stress and 1 subsequent day of reoxygenation cause the immunoreactivity of tyrosine hydroxylase (TH) and neuropeptide Y (NPY) to change, suggesting that these changes may be associated with adaptive responses, which are presumably employed to avoid oxidative damage and provide the initial mechanism for survival. Caspase-3 immunoreactive neurons increased eight-fold in the brain and 7.2-fold in the VNC after 1 day of reoxygenation, and the TUNEL-positive cell percentage rose from 0% (control) to 8.4% in the brain and from 1.7% (control) to 13% in the VNC. The results of our study provide evidence that anoxia and reoxygenation can activate caspase-3 and facilitate apoptosis in the CNS of crabs. These results provide evidence that even short-term air exposure stress followed by reoxygenation can trigger significant apoptotic cell death in crustacean neural tissue, which is important for developing better live transport practices.

## 1. Introduction

Air exposure stress during live transport from capture sites to markets is a common challenge for commercially valuable decapods such as crabs and lobsters. Prolonged exposure to air can result in significant water loss and hypoxia, which severely disrupt respiration, metabolism, locomotion, energetic homeostasis, behavior, and overall physiology, ultimately reducing survival rates [1,2]. Furthermore, reoxygenation following hypoxic stress during the recovery period can exacerbate physiological damage by increasing the production of reactive oxygen species (ROS), leading to oxidative stress and associated tissue damage [3,4,5].

The nervous tissue (brain and nerve cord) is among the most vulnerable to the damaging effects of ROS, because, under a lack of oxygen, the brain exhibits a high intensity of oxidative metabolic processes with low energy reserves and reduced antioxidant protection [6]. Crustaceans’ molecular mechanisms of response to hypoxia stress are known to be complex, with their regulation involving multiple genes and signaling pathways to provide survival [7]. This determines the fate of damaged neurons, triggering either pathological processes leading to cell death or compensatory adaptive mechanisms of recovery.

It has demonstrated that the changes in oxygen availability from hypoxia to reoxygenation lead to an increased level of ROS and risk of damage to various cell types via ROS-mediated apoptotic pathways [8,9].

To date, several studies have shown that hypoxia/anoxia and subsequent reoxygenation can induce apoptosis in crustacean peripheral organs and tissues, but their effects on neuronal apoptosis in crustaceans have not been studied [10,11]. In invertebrates, apoptosis caused by hypoxia, anoxia, and other injurious stimuli is primarily initiated in mitochondria through a process referred to as the intrinsic pathway [12], as the pro-apoptotic release of cytochrome c from mitochondria has been shown to be part of the endogenous apoptotic pathway in several invertebrates, including arthropods [13,14,15].

The central components of the apoptotic response are the activation of proteolytic enzymes named caspases [16]. Caspase-3 is considered a central regulator of apoptosis [16,17], since its activation leads to morphological alterations accompanying apoptosis [18].

Caspase-3 has been identified and characterized from mammals and many invertebrates, including crustaceans such as *Penaeus merguiensis* [19], *P. monodon* [20], *Litopenaeus vannamei* [21], *Eriocheir sinensis* [22], and *Scylla paramamosain* [23]. Although each species has unique duplications and diversities regarding the identified caspases, these specific caspases play similar roles and perform conserved functions [22]. As reported, caspase-3 activation in crustacean tissues is induced by a variety of injurious stimuli, such as heat, radiation, hypoxia/anoxia, and pathogenic bacterial and viral infections [22,23,24]. Furthermore, it has been demonstrated that caspase-3-like, at both the mRNA and protein levels, is involved in the apoptotic process after stimulation by different pathogen-associated molecular patterns in various tissues and in hemocytes [23]. Although the essential role of caspases in the apoptosis of crab neurons is well documented, the dynamics of caspase-3 activity in the CNS of crustaceans has not been systematically addressed to date.

One of the causes of adaptive and pathological neuron changes is the effect of hypoxia and reoxygenation on the metabolism of hormones and neurotransmitters [25].

Catecholamine metabolism might be especially crucially important for aquatic invertebrates during hypoxia and subsequent reoxygenation because tyrosine hydroxylase (TH), which is rate-limiting for the synthesis of the catecholamine’s enzymes, requires oxygen; thus, oxygen availability may limit the synthesis of these neurotransmitters. On the other hand, increased oxygen levels induce DA oxidation, thus triggering the generation of ROS. DA is a neurotransmitter under physiological conditions that may also serve as a neurotoxin and thereby participate in the apoptosis of neurons in vertebrates [26].

Tyrosine hydroxylase (TH) and dopamine (DA) synthesizing neurons have been well characterized in both the central and peripheral nervous systems in many crustacean species [27,28,29,30]. The involvement of biogenic amines in stressful environments has been demonstrated in several crustacean species, in which changes in hemolymphs under the influence of various unfavorable factors have been shown [31,32,33,34,35]. Several studies have also reported the involvement of DA in response to stress caused by hypoxia [36,37] in crustaceans. Moreover, researchers report the role of DA, which is involved in the regulation of ventilation [38], cardiac output [39], and glucose [40], in the hypoxia stress response of crustaceans. However, changes in dopamine signaling in the CNS that occur during hypoxia and reoxygenation in aquatic invertebrates have been scarcely studied. At the same time, it is known that, in vertebrates, hypoxia and reoxygenation cause damage to and the death of dopaminergic neurons [26,41,42].

Neuropeptide Y (NPY) is an important regulator in the response to hypoxia. It is one of the highly conserved signaling systems among metazoans that is involved in the suppression of apoptosis in mammalians [43,44]. NPY regulates a multitude of physiological and pathophysiological processes, including feeding/metabolic regulation, and is also an important stress resilience hormone [43,44,45]. In addition, NPY has also been observed to influence cardiovascular and respiratory responses to hypoxia [45,46,47] and may play a role in the mechanisms responsible for induced tolerance to hypoxia [47].

In invertebrates, neuropeptide Fs (NPFs) is an ortholog of NPY, which has a C-terminal amidated phenylalanine instead of the amidated tyrosine in vertebrates [28,48,49]. It has been noted that antibodies generated against vertebrate NPY family members produce labeling in the nervous system, as well as in other tissues, of invertebrates (Schoofs et al., 1988) [50]. NPFs have been identified in all crustacean species studied [28,48,49]. They have been implicated in modulating behavioral and sensory responses to stressful stimuli [51,52]. In crustaceans, NPFs play important roles in alleviating environmental stressors, such as ammonia [53], hypoxia [54,55,56], and low pH values [54]. Moreover, studies have shown increases in the brain levels of NPY homologs after hypoxia in crustaceans, which suggest their possible involvement in the stress response [55,56]. However, their role in air exposure and reoxygenation has not been fully determined.

The present study was designed to investigate the dynamics of the changes in DA and NPY signaling pathways in the CNS and their role in neuroprotective reactions and pathological processes that lead to cell death after air exposure and reoxygenation in crustaceans.

## 2. Materials and Methods

### 2.1. Experimental Animals

Adult male crabs, *Erimacrus isenbeckii* (Cheiragonidae, Decapoda), with a carapace width of 85 ± 6.7 mm (mean ± standard error of the mean) were captured in Peter the Great Bay, Sea of Japan.

After being transported to the laboratory, the crabs were placed in plastic tanks (140 L) with constantly aerated natural seawater and held for 1 week under the following normoxic conditions for acclimation: a dissolved oxygen concentration of 6.5 ± 0.2 mg/L, a water temperature of 3 ± 0.5 °C, a salinity of 32‰, and a natural photoperiod of 12 h/12 h (light/dark). The crabs were fed walleye pollock once a day.

### 2.2. Experimental Procedures

After acclimation, the 52 crabs were selected randomly and divided into the experimental and control groups. The crabs in the experimental group (32 crabs) were divided into subgroups of four crabs each and placed in standard Styrofoam boxes (78 × 39 × 23 cm) with freezer packs for 1 day (24 h) at 3 ± 0.5 °C. The crabs in the control group (20 crabs) were kept in aerated seawater throughout the experiment period under normoxic conditions as in the acclimation period. After 1 day (24 h) of air exposure, three crabs from the experimental and control groups were randomly selected and immediately dissected.

The other crabs from the experimental group were placed in plastic tanks with constantly aerated natural seawater under the same conditions as in the acclimation period. The crabs from these groups were selected for study 1, 7, and 14 days after reoxygenation. Control and experimental animals were analyzed simultaneously. Thus, we sampled crabs at the beginning of the experiment as a starting control (C); then after 24 h of air exposure stress, which was recorded as day 1 (A1); and then at 1 (R1), 7 (R7), and 14 (R14) days of reoxygenation.

### 2.3. Tissue Collection for Immunohistochemistry

The brain and ventral nerve cord (VNC) of control and experimental crabs was rapidly dissected after experiment. Nervous tissue was fixed in 4% formalin for 4 h at 4 °C. After several washes in PBS, the samples were embedded in the optimum cutting temperature medium Cryomount, frozen, and cut into 35–40 μm sections using a Cryo-Star HM560 MV cryostat (Thermo Fisher Scientific, Waltham, MA, USA). Sections (35–40 μm) were mounted on slides, coated with poly-L-lysine (Sigma-Aldrich; Spruce Street, Saint Louis, MO, USA), air-dried, and stored at −20 °C for subsequent staining. To eliminate nonspecific binding, the slides were incubated overnight in a blocking buffer comprising 10% normal donkey serum, 1% Triton-X 100, and 1% bovine serum albumin (BSA; Millipore, Burlington, MA, USA) dissolved in PBS at 4 C. The sections were then incubated overnight at 4 °C with a primary mouse anti-tyrosine hydroxylase antibody (1:100; Immuno Star, Hudson, WI, USA, #22941), rabbit anti-NPY polyclonal antibody (1:500; Sigma-Aldrich, Saint Louis, MO, USA #N9528), or rabbit anti-caspase-3 antibody (1:400) (Cell Signaling Technology, Inc., Danvers, MA, USA, #9661). After washing them three times with 0.01 M PBS (pH 7.4) containing 0.5% Triton X-100 (pH 7.4), the sections were incubated with 488-, 555-, or 647-Alexa Fluor conjugated donkey secondary antibodies (1:1000; Invitrogen, Thermo Fisher Scientific, Waltham, MA, USA) along with the nuclear marker DAPI (Sigma-Aldrich; Millipore, Burlington, MA, USA) for 2 h at 22° C. The sections were then washed and embedded in glycerol (Sigma-Aldrich; Millipore, Burlington, MA, USA).

### 2.4. Terminal Deoxynucleotidyl Transferase dUTP Nick End Labelling (TUNEL) Staining

TUNEL staining was performed using the TUNEL Andy Fluor™ 594 Apoptosis Detection Kit according to the manufacturer’s protocol (ABP Biosciences, #A050). However, all staining steps were performed on sections attached to glass slides. The TdT reaction was performed for 1 h at 37 °C in a humidified chamber. Finally, the cells were stained with DAPI for 5 min. The fluorescence signal was examined, and photographs were taken using a Zeiss LSM 700 confocal microscope.

### 2.5. Microscopy and Imaging

A Zeiss LSM 700 laser scanning confocal microscope was used to examined and capture images at the Far Eastern Center of Electron Microscopy, A.V. Zhirmunsky National Scientific Center of Marine Biology, Far Eastern Branch, Russian Academy of Sciences, Vladivostok, Russia. Following that, the images were exported from the confocal system as TIFF files. All images were processed and analyzed using ImageJ 1.53 (National Institutes of Health, Bethesda, MD, USA) software and the Imaris (Bitplane, Zurich, Switzerland). The presented figures show the projections of maximum immunoreactivity.

### 2.6. Quantification and Statistical Analysis

To quantify fluorescence, images of crabs’ brains and VNC were taken for the control and experimental groups (after 1 day of air exposure and 1, 7, and 14 days of reoxygenation) with the same scan settings. For fluorescence intensity quantification, images were processed using ImageJ 1.51w image processing software [57,58]. The quantitative evaluations measured the fluorescence of the expressed specific molecular markers in six whole cross-sections of the brain for all crabs and of the VNC for three control crabs and three experimental crabs (biological *n* = 3, technical *n* = 6) after 1 day of air exposure and 1, 7, and 14 days of reoxygenation.

Each image was imported into ImageJ (version 1.53, USA) and adjusted to the threshold using the method of Otsu (1979) [59], and the area and mean fluorescence of the foreground and the background along the entire image were measured. The corrected fluorescence was calculated according to the formula: Corrected total cell fluorescence (CTCF) = mean fluorescence of the foreground—(area of the foreground × mean fluorescence of the background).

For quantification of caspase-3- and TUNEL-positive nuclei, images were taken at 20× magnification. The values were obtained by counting the total cell number using a nuclear counterstain (DAPI) and the number of TUNEL- or caspase-3-positive cells. The caspase-3, TUNEL-, and DAPI-positive nuclei were measured in six whole cross-sections of the brain and VNC for three control crabs and three experimental crabs (biological *n* = 3, technical *n* = 6) after 1 day of air exposure and 1, 7, and 14 days of reoxygenation. The caspase-3- and TUNEL-positive nuclei were counted using ImageJ cell counter and are expressed as percentage out of the total number of DAPI-positive nuclei.

All data were analyzed and figures were prepared using Prism 7 software (GraphPad, San Diego, CA, USA). All measures for air exposure and control data were square-root transformed before statistical analysis to obtain normality and variance homogeneity. The caspase-3- and TUNEL-positive cell counts as well as CTCF from both experimental and control crabs were analyzed using a one-way analysis of variance (ANOVA) with Dunnett’s post hoc test or one-way ANOVA with Tukey’s multiple comparison tests. The results are presented as the mean ± standard error of the mean for each brain and VNC for each type of staining. Differences were considered significant at *p* < 0.05 and *n* = 6.

### 2.7. Neuroanatomical Nomenclature

The nomenclature used here to describe the anatomy, neuronal cell clusters, neuropils and regions of the brain and VNC of E. isenbeckii, refers to the classification system of previous studies, which has been used to describe several decapod species [60]

## 3. Results

### 3.1. Effects of Air Exposure and Reoxygenation on Survival Rate

**Figure 1:**
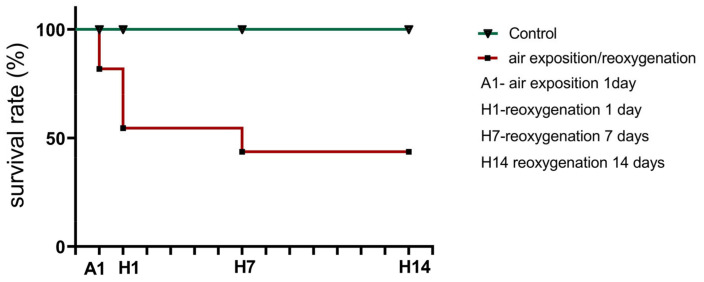
Kaplan–Meier survival curves of crabs, *Erimacrus isenbeckii*, in the control group (normoxia) and after experimental air exposure and reoxygenation (after 1, 7, and 14 days). Circles indicate crabs after air exposure and reoxygenation; triangles, control crabs. Only the air exposure/reoxygenation group showed mortality during the experimental period.

As shown by the analysis of the effects of air exposure and reoxygenation (Figure 1), there was no mortality in the control group. The onset of mortality was observed in the experimental crabs after 1 day of air exposure stress.

After 1 day of air exposure, most crabs exhibited slow eyestalk and antennae movements and dropped their legs and chelae without attempting to raise them. After 1 day of reoxygenation, the crabs were more active, and the movements of their legs and antennae were more evident compared to animals exposed to air for 1 day. When taken from the water for sampling, the individuals of the control group reacted with stronger resistance and had faster movements of their claws and legs compared to animals after 1 day of reoxygenation. After 7 days of reoxygenation, the recovery of their locomotor activity to the baseline level was observed. The survival rate analysis showed that the onset of the crabs’ mortality was after 1 day of air exposure and continued after reoxygenation (*p* < 0.001; Figure 1).

### 3.2. Effects of Air Exposure and Reoxygenation on Tyrosine Hydroxylase-like and Neuropeptide Y-like Immunoreactivity

The CNS in *E. isenbeckii*, as in other brachyuran crabs, consists of a brain (supraesophageal ganglion) and a ventral nerve cord (VNC), including the fused suboesophageal (SEG), thoracic (TG), and abdominal ganglia (AG).

The brain consists of three main parts: a protocerebrum (including optic ganglia, a lateral protocerebrum, and a median protocerebrum), a deutocerebrum, and a tritocerebrum. When describing the brain of *E. isenbeckii*, we focused only on the median brain (median protocerebrum, deutocerebrum, and tritocerebrum) and did not consider the lateral protocerebrum and the optic ganglia located in the eyestalks.

In our description based on double immunostaining for NPY and TH, we classified the neuronal cells of the brain and the VNC into size groups depending on their diameters: small (8–15 μm in diameter), medium-sized (21–40 μm), large (45–70 μm), and giant (80–120 μm).

**Figure 2:**
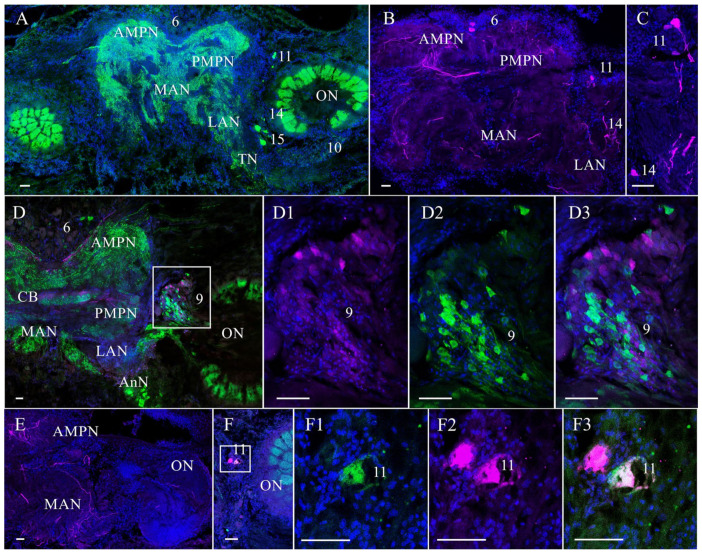
Distribution of neuropeptide Y (NPY)-like and tyrosine hydroxylase (TH)-like immunoreactivity in the median brain of control crabs, *E. isenbeckii*. (**A**). Horizontal sections through mid-ventral planes of the brain showing NPY-lir in AMPN, PMPN, LAN, MAN, and in neuronal clusters 11 and 14/15 (**B**). TH-lir in neuronal clusters 6, 11, and 14 and in AMPN, PMPN, LAN, and MAN (**C**). Neuronal clusters 11 and 14 including medium-sized neurons with high TH-lir (**D**). Horizontal sections through mid-ventral planes of the brain showing NPY- and TH-lir in AMPN, PMPN, CB, LAN, MAN, ON, and AnN and in clusters 6 and 9 (**D1**–**D3**). Double-labeling for NPY (green) and TH (magenta) in cell cluster 9 (**E**). Immunolocalization of TH-lir in the proto- and deutocerebrum (**F**–**F3**). Immunodetection of TH-lir (magenta) and its colocalization with NPY-lir (green) in cell cluster 11. The letter designations are as follows: AMPN, anterior medial protocerebral neuropil; PMPN, posterior medial protocerebral neuropil; ON, olfactory neuropil; CB, central body; LAN, lateral antenna l neuropil; MAN, medial antenna l neuropil; AnN, antenna II neuropil; 6, 9 and 11, 14/15 are cell clusters. Color designations: magenta indicates TH; green, NPY; blue, DAPI. Scale bars: 100 μm.

**Figure 3:**
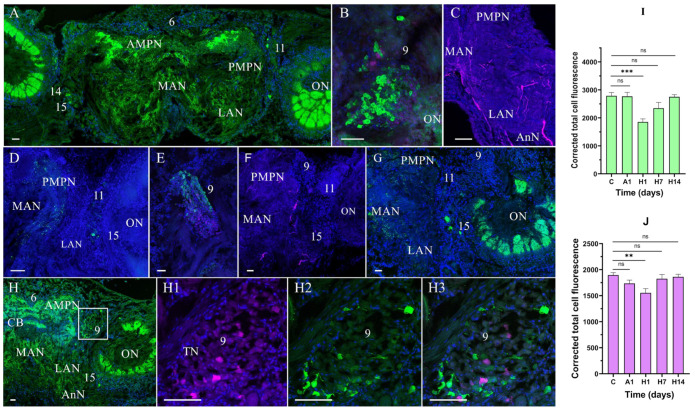
Changes in neuropeptide Y (NPY)-like and tyrosine hydroxylase (TH)-like immunoreactivity in the median brain of crabs, *E. isenbeckii*, after 1 day of air exposure (**A**–**C**) and reoxygenation at 1 (**D**–**F**), 7 (**G**), and 14 (**H**–**H3**) days. High NPY-lir in fibers in AMPN and ON (**A**). NPY- and TH-lir in neurons of cell cluster 9 (**B**). TH-lir in fibers in AnN (**C**). A decrease in NPY-lir in AMPN, PMPN, MAN, LAN, and ON at 1 day of reoxygenation (**D**). A decrease in NPY- and TH-lir in neurons of cell cluster 9 (**E**). TH-lir in fibers of PMPN and MAN (**F**). NPY-lir in PMPN, MAN, LAN, and ON at 7 days of reoxygenation (**G**). NPY-lir in AMPN, PMPN, MAN, LAN, and ON at 14 days of reoxygenation (**H**). Immunodetection of TH- and NPY-lir and their colocalization in cell cluster 9 (**H1**–**H3**). Changes in signal intensity of NPY- (**I**) and TH-lir (**J**) in the median brain after air exposure and reoxygenation (**I**). The letter designations are as follows: AMPN, anterior medial protocerebral neuropil; PMPN, posterior medial protocerebral neuropil; ON, olfactory neuropil; CB, central body; LAN, lateral antenna I neuropils; MAN, medial antenna l neuropils; 6, 9, and 11 are cell clusters. Color designations: magenta indicates TH; green, NPY; blue, DAPI. Scale bars: 100 μm. Data were analyzed by one-way ANOVA followed by Dunnett’s multiple comparison test in GraphPad Prism 7. Data are presented as mean ± standard error of the mean (*n* = 6); ns, no significance; ** *p* < 0.01; *** *p* < 0.001.

**Figure 4:**
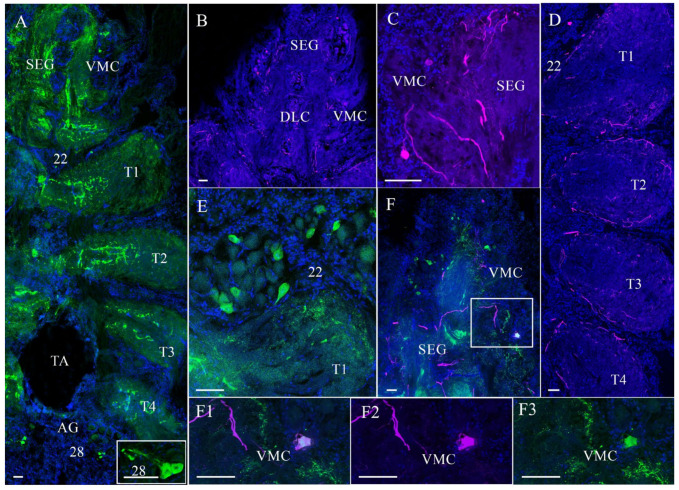
Distribution of neuropeptide Y (NPY)-like and tyrosine hydroxylase (TH)-like immunoreactivity in the ventral nerve cord (VNC) of control crabs, *E. isenbeckii.* Horizontal sections through the VNC showing NPY-lir in the suboesophageal ganglion (SEG), thoracic ganglia (TG), and abdominal ganglion (AG) (**A**). A part of the SEG showing TH-lir in dorsolateral clusters (DLC) and ventro-medial cluster (VMC) (**B**). A part of the SEG showing TH-lir neurons in VMC (**C**). TH-lir fibers in neuropils of TG (**D**). NPY-lir neurons in cell cluster 22 (**E**). Combined figure illustrating the distribution of NPY- and TH-lir in the SEG (**F**). Double-labeling for NPY- (green) and TH-lir (magenta) in neurons of VMC (**F1**–**F3**). The letter designations are as follows: SEG, suboesophageal ganglion; TG, thoracic ganglion; TA, thoracic artery; T1–T5, neuropils of TG; DLC, dorsolateral cluster; VMC, ventro-medial cluster; AG, abdominal ganglion; 22 and 28 are cell clusters. Color designations: magenta indicates TH; green, NPY; blue, DAPI. Scale bars: 100 μm.

**Figure 5:**
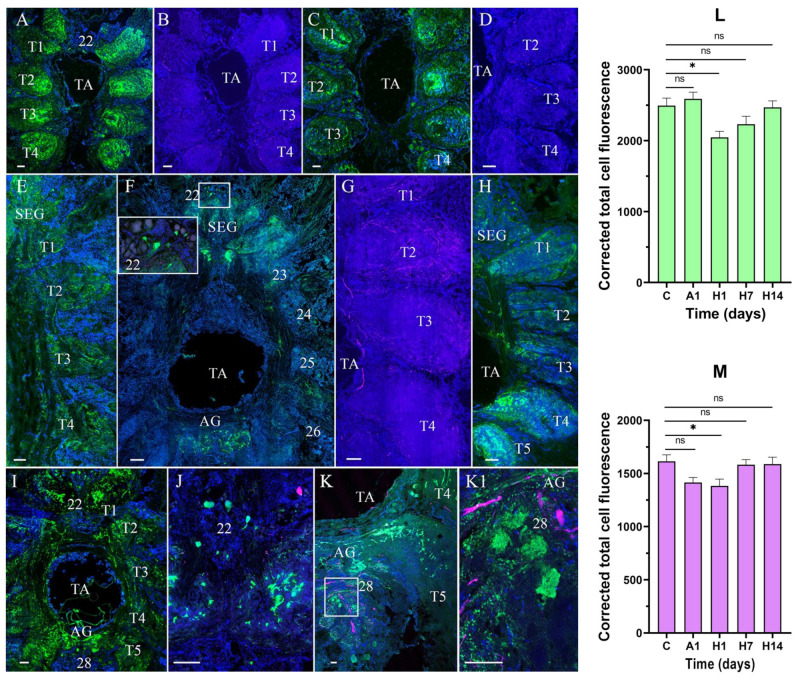
Changes in neuropeptide Y (NPY)-like and tyrosine hydroxylase (TH)-like immunoreactivity in the ventral nerve cord (VNC) of crabs, *E. isenbeckii*, after 1 day of air exposure (**A**,**B**) and reoxygenation at 1 (**C**,**D**), 7 (**E**–**G**), and 14 (**H**–**K1**) days. Mid-ventral section showing increase in NPY-lir in neuropils of thoracic ganglia (TG) (T1–T5) (**A**). Mid-ventral section showing decrease in TH-lir in TG (**B**). Decrease in NPY-lir in neuropil of TG (**C**). TH-lir in nerve fibers in TG (**D**). NPY-lir in SEG and TG after 1 day of reoxygenation (**E**). NPY-lir in cell cluster 22 (**F**). Increase in TH-lir in TG after 7 days of reoxygenation (**G**). Increase in NPY-lir in TG after 14 days of reoxygenation (**H**). Dorsal section showing NPY-lir in TG and AG (**I**). NPY-lir neurons in cell cluster 22 (**J**). Increase in NPY-lir in neurons and nerve fibers in the AG after 14 days of reoxygenation (**K**). Expression of NPY-lir in cell cluster 28 (**K**,**K1**). Changes in signal intensity of NPY- and TH-lir in VNC after air exposure and reoxygenation (**L**,**M**). The letter designations are as follows: SEG, suboesophageal ganglion; TA, thoracic artery; T1–T5, neuropils of TG; AG, abdominal ganglion; 22 and 28 are cell clusters. Color designations: magenta indicates TH; green, NPY; blue, DAPI. Scale bars: 100 μm. Data were analyzed by one-way ANOVA followed by Dunnett’s multiple comparison test in GraphPad Prism 7. Data are presented as mean ± standard error of the mean (*n* = 5); ns, no significance; * *p* < 0.05.

In the control and experimental crabs, tyrosine hydroxylase-like (TH-lir) and neuropeptide Y-like immunoreactivities (NPY-lir) were detected in cell clusters and numerous fibers of all the median brain and VNC regions (Figure 2, Figure 3, Figure 4 and Figure 5).

In the median protocerebrum of the control crabs, TH- and NPY-lir were localized in the anterior and posterior medial neuropils (AMPN and PMPN) and the central body (CB) (Figure 2A,B,D,E). TH- and NPY-lir were also detected in small- and medium-sized neurons of cluster 6 (Figure 2B,D).

In the deutocerebrum, TH- and NPY-lir were observed in the lateral antenna I neuropil (LAN) and the medial antenna I neuropil (MAN) (Figure 2A,B,D,E). The paired olfactory neuropil (ON) displayed intense NPY-lir (Figure 2A,D,F), but the expression of TH-lir in the ON was very weak and barely detectable (Figure 2E). In this region, a population of NPY- and TH-lir neurons was detected in cell clusters 9 and 11 (Figure 2A–D3,F–F3). In cluster 9, there were small (12–18 µm in diameter) globular NPY- and TH-lir neurons, which were local olfactory interneurons. With double immunolabeling, the colocalization of NPY- and TH-lir was not observed in these neurons (Figure 2D–D3). Cluster 11 contained medium-sized and small NPY-lir (Figure 2A,F,F1,F3) and also medium-sized TH-lir neurons (Figure 2B,C,F,F2,F3). Double immunolabeling revealed the presence of single medium-sized neurons of this cluster that expressed both NPY- and TH-lir (Figure 2F–F3).

In the tritocerebrum, NPY- and TH-lir were also observed in the antenna II (AnN) and tegumentary neuropils (TN) (Figure 2A,D). In addition, NPY- and TH-lir were also found in single small- and medium-sized neurons of clusters 14/15 (Figure 2A–C).

In the experimental crabs, the distribution patterns of both neurotransmitters were similar after hypoxia and reoxygenation; however, the intensities of NPY- and TH-lir differed.

In the median brain of the crabs exposed to air for 1 day (24 h), the expression of NPY-lir was high in the AMPN, ON, and cluster 9 cells, but no apparent differences were observed in NPY-lir in other regions and cell clusters compared to the control group (Figure 3A,B). Moreover, after 1 day of air exposure, TH-lir was found to insignificantly decrease in the median protocerebrum; in the tritocerebrum, varicose nerve fibers, however, showed more intense TH-lir than that of the control (Figure 3C).

After 1 day of reoxygenation, there was a significant decrease in NPY-lir in the nerve fibers and cell clusters in all regions of the median brain compared to that after 1 day of air exposure (Figure 3D,E). TH-lir decreased in all regions of the brain (median protocerebrum, deutocerebrum, and tritocerebrum) after 1 day of reoxygenation and was restored to the control level after 7 days of reoxygenation (Figure 3E,F).

In the experimental crabs exposed to 7 days of reoxygenation, the CSLM image analysis showed that the NPY-lir of all regions of the brains was not fully restored compared to the normoxic control (Figure 3G). In addition, individual NPY-lir variability was observed in some of the crabs after 1 day of air exposure and 1 day of reoxygenation.

A quantitative analysis of corrected total cell fluorescence (CTCF) showed a decrease in the NPY-lir signal in the median brain after 1 day of reoxygenation (to 1854.73 ± 89.58, *p* < 0.001), compared to the respective control values (2786.28 ± 119.1) (Figure 3I). Furthermore, after 1 day of air exposure and 1 day of reoxygenation, the TH-lir intensity decreased in the median brain (1735.44 ± 65.42, *p* > 0.05, and 1554.44 ± 81.09, *p* < 0.01, respectively) compared to that in the control (1895.75 ± 47.2) (Figure 3J). In the brains of the crabs exposed to 14 days of reoxygenation, NPY-lir was restored to the control level, but there was a decrease in NPY-lir in cluster 9 cells (Figure 3H–H3).

In the VNC of the control and experimental crabs, TH- and NPY-lir were detected in the suboesophageal (SEG), thoracic (TG), and abdominal ganglia (AG) (Figure 4A–F3).

In the SEG, NPY-lir was detected in medium-sized and small neurons in ventromedial clusters (VMCs) (Figure 4A,F,F1,F3). In the SEG, single medium-sized neurons expressing TH-lir were also present in both the dorsolateral clusters (DLC) and VMC (Figure 4B,C,F,F1,F2). The colocalization of NPY- and TH-lir was observed only in certain medium-sized neurons of DLC (Figure 4F–F3). The TG contained single TH- and NPY-lir neurons in cell clusters 22–27 and TH- and NPY-lir-positive fibers in all the thoracic neuropils (Figure 4A,D). In the AG, cells of cluster 28 contained predominantly medium-sized cells expressing NPY- and TH-lir, as well as NPY-lir fibers (Figure 4A).

In the VNC, exposure to anoxia for 1 day caused the increased expression of NPY-lir in the neuropils of the TG relative to the control (Figure 5A). Simultaneously, after 1 day of anoxia, a decrease in TH-lir was observed in all regions of the VNC compared to the control (Figure 5B).

After 1 and 7 days of reoxygenation, a decrease in the expression of NPY-lir was detected in the SEG, TG, and AG relative to the control (Figure 5C,E,F). The intensity of TH-lir also decreased after 1 day of reoxygenation (Figure 5D). However, after 7 days of reoxygenation, the expression of TH-lir had a similar level to that of the normoxic control (Figure 5G).

After 14 days of reoxygenation, NPY-lir was higher in the neuropils of the TG and AG than after 7 days of reoxygenation (Figure 5H–K1). In addition, there was an increase in NPY-lir in the neurons of the SEG (Figure 5I,J) and in those of cluster 28 in the AG (Figure 5K,K1). NPY- and TH-lir were not colocalized in these neurons.

A quantitative analysis of NPY-lir intensity showed a decrease compared to the control (2493.65 ± 106.15) after 1 day (2046.96 ± 84.19, *p* < 0.05) and 7 days (2231.21 ± 113.79, *p* > 0.05) of reoxygenation (Figure 5L). Moreover, a decrease in TH-lir signal was found in the VNC after 1 day of air exposure (1414.86 ±47.19, *p* > 0.05) and 1 day of reoxygenation (1382.54 ± 64.75, *p* < 0.05) compared to the control (1615.1 ± 60.92) (Figure 5M).

### 3.3. Effects of Air Exposure and Reoxygenation on Cell Death

**Figure 6:**
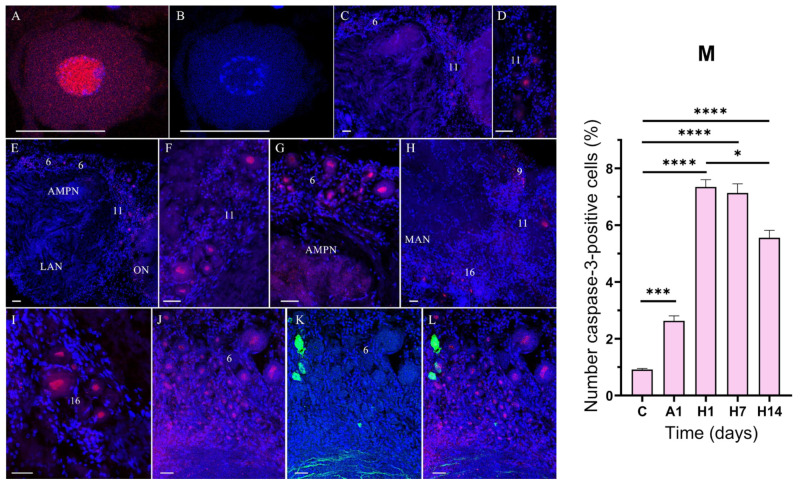
Changes in caspase-3-lir in the median brain of crabs, *E. isenbeckii*, in control (**C**,**D**) and after 1 day of air exposure (**E**,**F**) and reoxygenation at 1 (**G**,**H**), 7 (**H**,**I**), and 14 (**J**–**L**) days. Caspase-3-lir neuron (**A**). DAPI staining of nuclei (**B**). Horizontal sections through mid-ventral planes of a part of the brain showing single caspase-3-lir neurons in clusters 6 and 11 (**C**). Caspase-3-lir neurons in cluster 11 (**D**). Increase in caspase-3 expression in clusters 6 and 11 after 1 day of air exposure (**E**). Expression of caspase-3 in neurons in clusters 11 (**F**). Increase in caspase-3 expression in cluster 6 after 1 day of air exposure (**G**). Caspase-3-lir neurons in clusters 9, 11, and 16 (**H**). Caspase-3-lir neurons in cluster 16 (**I**). Double-labeling for TH (green) and caspase-3 (red) in neurons of cluster 6 (**J**–**L**). Numbers of caspase-3-positive cells in different median brain regions in control and after air exposure and reoxygenation (**M**). The letter designations are as follows: AMPN, anterior medial protocerebral neuropil; ON, olfactory neuropil; LAN, lateral antenna I neuropils; MAN, medial antenna l neuropils; 6, 9, 11, and 16 are cell clusters. Color designations: red indicates caspase-3; green, TH; blue, DAPI. Scale bars: 50 μm. Data were analyzed by one-way ANOVA followed by Dunnett’s multiple comparison test in GraphPad Prism 7. Data are presented as mean ± standard error of the mean (*n* = 6); ns, no significance; * *p* < 0.05; *** *p* < 0.001; **** *p* < 0.001.

To determine the molecular basis of the air-exposure- and reoxygenation-induced death of neurons, the brain and VNC of *E. isenbeckii* were subjected to caspase-3 immunostaining, DNA labeling with DAPI, and TUNEL staining (Figure 6A,B). The proportion of TUNEL- and caspase-3-labeled cells was estimated for the median brain and VNC.

**Figure 7:**
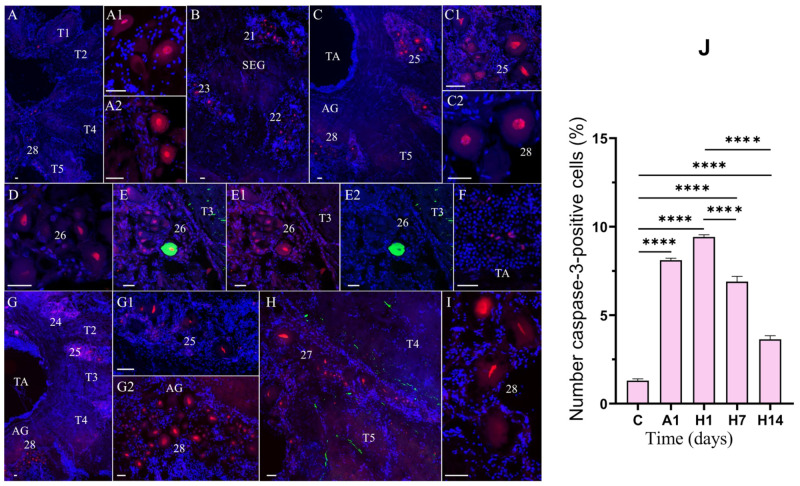
Changes in caspase-3-lir in the ventral nerve cord (VNC) of crabs, *E. isenbeckii*, in control (**A**–**A2**) and after 1 day of air exposure (**B**–**C2**) and reoxygenation at 1 (**D**–**F**), 7 (**G**–**G2**), and 14 (**H**,**I**) days. Caspase-3-lir neurons in TG of control crabs (**A**). Presence of caspase-3-lir in cell cluster 28 (**A1**). Caspase-3-lir neurons in cell cluster 26 (**A2**). Expression of caspase-3 in cell clusters 21 (ventro-medial cluster, VMC), 22, and 23 (**B**). Caspase-3-lir neurons in cell clusters of TG (**C**). Numerous caspase-3-lir neurons in cell cluster 25 (**C1**). Caspase-3-lir neurons in cell cluster 28 of AG (**C2**). Caspase-3-lir neurons in cell cluster 26 of TG (**D**). Immunolabeling for TH (green) and caspase-3 (red); combined figure showing their colocalization in cell cluster 26 (**E**–**E2**). Caspase-3-lir hemocytes in thoracic artery (TA) (**F**). Caspase-3-lir neurons in cell clusters of TG and AG after 7 days of reoxygenation (**G**). Caspase-3-lir neurons with altered nuclei in cell cluster 25 (**G1**). Numerous caspase-3-lir neurons in cell cluster 28 (**G2**). Caspase-3-lir neurons with altered nuclei in cell cluster 27 (red) and TH-lir nervous fibers (green) (**H**). Caspase-3-lir neurons in cell cluster 28 after 14 days of reoxygenation (**I**). Quantification for caspase-3 neurons in cell clusters in VNC of crabs, *E. isenbeckii*, in control, after 1 day of air exposure and at 1, 7, and 14 days of reoxygenation (**J**). The letter designations are as follows: SEG, suboesophageal ganglion; TA, thoracic artery; T1–T5, neuropils of TG; AG, abdominal ganglion; 21, 22, 23, 24, 25, 26, 27, and 28 are cell clusters. Color designations: red indicates caspase-3; green, TH; blue, DAPI. Scale bars: 50 μm. Data were analyzed by analyzed using a one-way analysis of variance (ANOVA) with Dunnett’s post hoc test or one-way ANOVA with Tukey’s multiple comparison tests. Data are presented as mean ± standard error of the mean (*n* = 6); **** *p* < 0.0001.

In the median brain and VNC, caspase-3-like immunoreactivity (caspase-3-lir) was observed in cell nuclei (Figure 6A,C–L and Figure 7A–I). In the median brain of the control crabs, caspase-3-lir was detected in single neurons in clusters 6 and 11 (Figure 6C,D). After 1 day of air exposure, the expression of caspase-3 increased in cell clusters 6 and 11 (Figure 6E–G).

After 1 day of reoxygenation, the expression of caspase-3 was also found in cells of clusters 9 and 16 (Figure 6H,I). Subsequently, after 1 day of reoxygenation, the expression of caspase-3 markedly increased only in the cells of cluster 6 and remained at a high expression level at 7 days of reoxygenation compared to the control (Figure 6J,L). In this cell cluster, we also observed neurons in which caspase-3 was coexpressed with TH (Figure 6J–L).

In the VNC of the control crabs, caspase-3-lir was visible in the nuclei of single neurons in several cell groups in the TG and AG (Figure 7A–A2). After 1 day of air exposure, caspase-3 expression in neurons was detected in the SEG, TG, and AG (Figure 7B–C2). The greatest proportion of caspase-3 cells was recorded from cell clusters 21, 22 (Figure 7B), and 28 (Figure 7C). Significant individual variability was found in the number of caspase-3-lir neurons in clusters 23–27 in the TG (Figure 7D–E1). In these cell clusters, we also observed neurons in which caspase-3 was coexpressed with TH (Figure 7E–E2). In addition, expression of caspase-3 in hemocytes was observed after air exposure (Figure 7F). In all VNC regions, the expression of caspase-3 was upregulated after 1 day of reoxygenation, but after 7 days of reoxygenation, its expression level decreased (Figure 7 G,G1) except in cluster 28 in the AG (Figure 7G2). After 14 days of reoxygenation, the number of caspase-3-lir cells decreased in many cell clusters of the VNC (Figure 7H,I).

As shown by the quantitative analysis (Figure 6M and Figure 7J), in the control crabs, very few neurons in the median brain and VNC showed caspase-3-lir in their nuclei, indicating minimal active apoptosis under normal conditions. After 1 day of air exposure, an increased number of neurons displayed caspase-3-lir (Figure 6M and Figure 7J), which was more significant in all areas of the VNC (the SEG, TG, and AG) (Figure 7B–C2,J). Following 1 day of reoxygenation, caspase-3-lir became even more widespread, with significantly more caspase-3-positive neurons observed (Figure 6M and Figure 7J). Quantitatively, the proportion of caspase-3-lir neurons in the median brain rose from 0.9% in the controls to 7.3% after 1 day of reoxygenation (Figure 6M; *p* < 0.0001 vs. control) and in the VNC from 1.3% in the controls to 9.4% after 1 day of reoxygenation (Figure 7J; *p* < 0.001 vs. control). After a prolonged recovery, the number of caspase-3-lir cells decreased again. After 7 days of reoxygenation, there were fewer caspase-3-lir cells, and after 14 days, they had significantly decreased compared to the 1-day reoxygenation peak (Figure 6M, ~5% after 14 days vs. ~7% after 1 day of reoxygenation in the median brain, *p* < 0.01; Figure 7J, 3.6% after 14 days vs. 9.4% after 1 day of reoxygenation in the VNC, *p* < 0.0001). This indicates recovery from the apoptotic wave.

**Figure 8:**
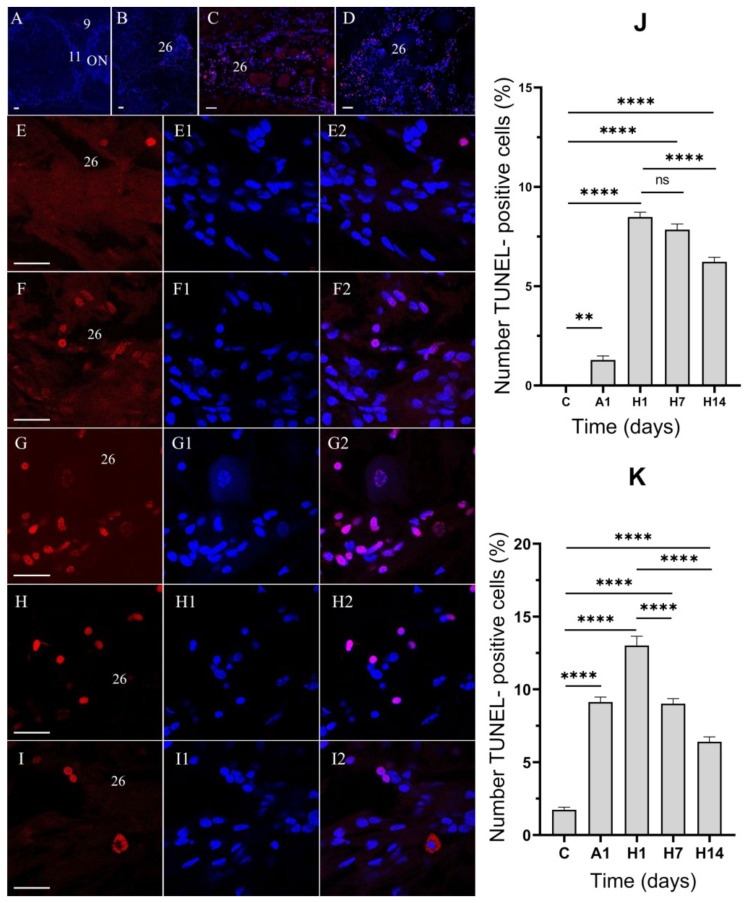
TUNEL-positive cells in the brain (**A**) and ventral nerve cord (VNC) (**B**–**I2**) of crabs, *E. isenbeckii*, in control, after 1 day of air exposure, and at 1, 7, and 14 days of reoxygenation. Horizontal sections through mid-ventral planes of a part of the brain showing single TUNEL-positive cells in cluster 9 after 1 day of anoxia (**A**). TUNEL-positive cells in cluster 26 of TG in control crabs (**B**), after 1 day of air exposure (**C**), and after 1 day of reoxygenation (**D**). TUNEL-positive cells in cluster 26 of TG in control (**E**–**E2**), after 1 day of air exposure (**F**–**F2**), and reoxygenation at 1d (**G**–**G2**), 7d (**H**–**H2**), and 14 days (**I**–**I2**). Cytoplasmic TUNEL signaling in cluster 26 of TG after 14 days of reoxygenation (**I**–**I2**). Quantitative assessment of TUNEL-positive cells in the brain and VNC of crabs, *E. isenbeckii*, in control, after 1 day of air exposure and at 1, 7, and14 days (**J**,**K**). The letter designations are as follows: AMPN, anterior medial protocerebral neuropil; ON, olfactory neuropil; cell clusters 9, 26. Red indicates TUNEL staining; blue, DAPI staining. Scale bars: A–D—50 μm; E–I2—25 μm. Data analysis was performed in GraphPad Prism 7 using a one-way analysis of variance (ANOVA) with Dunnett’s post hoc test or one-way ANOVA with Tukey’s multiple comparison tests. Data are presented as mean ± SEM (*n* = 5), ns, no significance, ** *p* < 0.01, **** *p* < 0.0001.

In the brains of the control crabs, no TUNEL-positive neurons were found, but TUNEL-positive cells were detected in the cell clusters of the VNC (Figure 8A). After 1 day of air exposure, TUNEL-positive apoptotic cells began to appear in cell cluster 9 of the median brain (Figure 8B). Numerous apoptotic cells were revealed in all clusters of the VNC after 1 day of air exposure (Figure 8C). After 1 day of reoxygenation, the number of apoptotic cells further increased in all regions of the brain and VNC (Figure 8D). Among the TUNEL-positive cells, hemocytes were revealed in all cell clusters in the TG and AG (Figure 8D–H2).

Consistent with the quantitative summary of the caspase-3 data, the quantitative TUNEL assay showed very few apoptotic nuclei in the CNS of the control crabs (Figure 8J,K; ~1% of cells in the VNC were TUNEL-positive). However, after 1 day of air exposure, there was an increase in the number of TUNEL-positive cells in the clusters of the median brain and VNC (Figure 8J,K). After 1 day of reoxygenation, there was a dramatic increase in TUNEL-positive nuclei in the median brain and a further increase in the number of apoptotic nuclei in the VNC (Figure 8J,K), indicating extensive DNA fragmentation. The proportion of TUNEL-positive cells rose to ~8% in the median brain after 1 day of reoxygenation (Figure 8J, *p* < 0.001 vs. control) and to ~13% in the VNC after 1 day of reoxygenation (Figure 8K, *p* < 0.001 vs. control). After 7 days of reoxygenation, TUNEL labeling remained elevated compared to the control (Figure 8; ~7% in the median brain and ~9% in the VNC), though slightly lower than that of the 1-day reoxygenation group, suggesting some recovery.

## 4. Discussion

Our present study showed that anoxia and the subsequent increase in O_2_ concentration (reoxygenation) alter DA- and NPY-lir, inducing caspase-3 expression and apoptosis in the CNS of horsehair crab, *Erimacrus isenbeckii*.

Changes in the activity of neurotransmitters and hormones in the central nervous system are an important and fundamental component of the response of crustacea to hypoxia.

NPY and DA are involved in pathway activation during stress and are involved in neuroprotection and neurodegeneration in vertebrates [26,44,45,61]. However, little is known about neurotransmitters and hormones in the central nervous system (CNS) induced under hypoxia and reoxygenation and their role in the resistance or death neurons of crustacea.

In the present study, we observed an insignificant upregulation of NPY-lir expression in certain areas of anterior medial protocerebral neuropils in the brain and in the thoracic ganglion of VNC in *E. isenbeckii* after 1 day of air exposure. In crustaceans, these anatomical regions have projections to the optic ganglia and lateral protocerebrum in the brain, which is associated with multimodal processing, and in the neuropils of the thoracic ganglion in the VNC, which are involved in sensorimotor integration and neuroendocrine regulation. The local expression of NPY-lir in the median brain and VNC of crabs after 1 day of anoxia suggests their involvement in the stress response. Previously, elevated NPY levels were shown to increase stress resistance in mammals [62] and insects [52]. Experiments on Drosophila have shown that NPY can mediate resistance to various stressors, including olfactory, temperature, mechanical stresses, etc. [52].

In the median brain and VNC of *E. isenbeckii,* NPY-lir colocalizes with TH-lir in some neurons. Previously, in larval *D. melanogaster,* NPF colocalization with most dopaminergic neurons was shown, and the authors suggested an extensive interplay between these two signaling pathways, which leads to the modulation of the perception of external stress stimuli in flies [52]. Эro agrees with the fact that NPY-like peptides modulate behavioral responses to key environmental parameters, including O_2_ level [54]. It was shown that in blue crabs (Callinectes sapidus), hypoxia induces variations in levels of RFamides, which are homologs to NPY in the crustacean brain, depending on hypoxia severity [55,56]. According to Buchberger et al., 2020, changes in NPY expression may help protect crabs from hypoxia through variations in heart rate [55,56]. As the authors suggest, these biochemical alterations are linked to the adaptative mechanisms that could provide an initial survival mechanism for hypoxia [55,56]. In crustaceans, NPY-like peptides are autocrine/paracrine modulators and hormones that can influence physiological parameters, such as ventilation and levels of glucose in hemolymphs, carbohydrate metabolism, and heart rate [27]. Under conditions of oxidative metabolism imbalance, they play a neuroprotective role in modulating neuronal physiology through the regulation of calcium homeostasis, neurotransmitter release, and synaptic excitability [63].

In our study, in the median brain and VNC, we recorded a decrease in NPY-lir after 1 day of reoxygenation in the recovery period when normalization could be expected. After 7 days of re-oxygenation, the expression of TH-lir was gradually restored but did not reach the initial levels, suggesting the negative effects of hypoxia. As previously shown, the dysregulation of NPY can lead to an increased vulnerability of neurons to subsequent stress or decrease stress resistance [45,52].

Besides the decreased NPY in the crabs that underwent air exposure and reoxygenation compared to the control, we observed a variation in DA expression. Dopamine (DA) is a neurotransmitter involved in oxygen sensing and the control of reflex hyperventilation in both vertebrates and invertebrates [38,64]. TH-lir decreased in the median brain and VNC of *E. isenbeckii* after 1 day of air exposure, which could indicate the release and transport of DA from nerve terminals to the peripheral organs and hemolymph in response to hypoxia. In support of this, earlier studies have shown that hypoxia significantly increases the concentration of dopamine (DA) in hemolymph in several species of crustaceans [36,37]. These data are consistent with the results of biochemical studies which have shown changes in the DA level and ratio in crustacea hemolymphs under the effects of other unfavorable stress factors [31,32,33,34,35]. A change in the concentration of DA in hemolymphs is a neuroendocrine response of invertebrates to stress that depends on the intensity and duration of the action stress factor and, in physiological conditions, allows for the metabolic and behavioral adaptation of these animals to adverse impacts [31,36,37]. It was found that DA modulates most cardiorespiratory responses to hypoxia, with an effect on heart rate, ventricular pressure, and outflow through the sternal arterial valve in crustaceans [39]. Additionally, it stimulates the rhythmic beating of the scaphognathites in vitro at the level of ventilatory pattern generator neurons [38] and regulates the ventilation rate [2]. Furthermore, DA changes glucose levels during hypoxia [40]. In *E. isenbeckii* after 1 day of reoxygenation, TH-lir did not recover and returned to normal only after 7 days of reoxygenation. The decrease in TH-lir in the CNS of crabs may be associated with increased ROS concentrations and reoxygenation. Furthermore, our study showed the expression of the proapoptotic gene caspase-3 in dopaminergic neurons, which was the highest after 1 day of preoxygenation. Elevated levels of ROS and reactive nitrogen species due to excess O_2_ supply play a significant role in the damage accrued due to reoxygenation. It is known that DA can be spontaneously oxidized to form reactive oxygen species (ROS), free radicals, and quinones [65].

These oxidation products can damage cellular components such as lipids, proteins, and DNA (14). DA-induced oxidative stress is considered to be a major pathological factor of apoptosis. This is due to the high level of exposure of these dopaminergic neurons to ROS, especially those formed by DA oxidation [26]. This is supported by studies showing that crab tissues have elevated oxidative stress indices during hypoxia and reoxygenation. O_2_ has a direct effect on mitochondrial function and may release mitochondrial cytochrome-c in cell cytosols and induce mitochondrial dysfunction in neurons [66]. Recent studies have shown that caspase-3 plays a central role in apoptotic cell death in invertebrates [22,23,24]. Caspase-3, regarded as a crucial protein of apoptosis, is constitutively expressed as an inactive precursor in cells [17]. During ischemia, caspase-3 is cleaved and activated whereupon it degrades multiple substrates in the cytoplasm and nucleus, leading to cell death. In our study, 1 day of exposure to anoxia induced caspase-3 activation in neuron and hemocyte nuclei in the CNS of horsehair crabs (*E. isenbeckii*), but after 1 day of reoxygenation, the number of caspase-3-immunoreactive cells increased in all regions of the CNS and was particularly pronounced in the VNC. These data are consistent with previous studies that showed the activation of caspase-3 in various cell types and tissues under hypoxia/anoxia in vertebrates and in various groups of invertebrates, including several species of crustaceans [22,23,24]. In mammals, caspase-3 activation was detected following ischemia to the brain, where it overlapped with the areas of increased apoptotic cell death [67]. To date, little is known about the involvement of caspase-dependent pathways in hypoxia-induced neuron apoptosis in crustaceans. However, there is evidence that air exposure for 12.5 h can lead to a significant increase in caspase-3 expression levels and to apoptosis in the hepatopancreas and gills of shrimp (*Marsupenaeus japonicus)* [10]. It has also been shown that other adverse stimuli, such as bacterial infections, can activate caspase-3 and trigger apoptosis in crabs [23,24,68]. In addition, knocking down Sp-caspase 3 in vivo significantly reduced cell apoptosis and increased the mortality of mud crabs from an infection caused by *Vibrio parahaemolyticus*. The authors hypothesized that caspase-3 played important roles in apoptosis against bacterial infection in crabs [23,24,68]. In the present study, the caspase-3 expression increased initially, and then, after 7 days of reoxygenation following anoxia, it decreased. Previous experiments on organs and tissues of mammals under conditions of ischemia-reperfusion showed that apoptotic signaling emerges after the chronic hypoxic stress because hypoxia inhibits the ability to produce ATP, the Na^+^ -K^+^ ion pump cannot be maintained, and the cells eventually depolarize and undergo necrosis and apoptosis [69]. However, according to recent studies, hypoxia or reoxygenation, or both, lead to increased levels of apoptosis and induce cell death shortly after reoxygenation in some fish species [42]. In crabs *(E. isenbeckii*), the caspase-3 activity was enhanced after hypoxia and reoxygenation, correlating with an increased death rate. To clarify whether caspase-3 activation was associated with the induction of apoptosis, the brains and VNC from experimental crabs were analyzed after detection via TUNEL staining. In our TUNEL assay, the apoptotic signaling began to emerge after 1 day of anoxia, and after 1 day of reoxygenation, the percentage of apoptotic cells in the CNS of the crabs increased. However, the underlying apoptotic signaling mechanisms were not sufficiently clarified. The fact that the numbers of dying cells in the brain and VNC were the highest after reoxygenation indicates that the cell death we observed was more likely induced by ROS than by the lack of oxygen. As previously shown, the ROS production increases during hypoxia and reoxygenation as a result of leakage from the electron transport chain and that such an increase in ROS can induce cell death [70]. According to the results of our experiments, the caspase-3 activation correlates with the induction of apoptosis in neurons. Our findings are consistent with previous studies on vertebrate and invertebrate species, in which hypoxia induced apoptosis with caspase-3 activation [10]. Similar results were obtained for the neurons of Ucides cordatus crabs upon ultraviolet irradiation [71]. The increase in TUNEL-positive cells was accompanied by an increase in the activity of caspase-3 cells, thus confirming that the DNA fragmentation observed via TUNEL labelling was a result of the activation of caspase-dependent apoptosis. The increase in the number of caspase-3-expressing cells in the brain and VNC was accompanied by a significant increase in the number of TUNEL-positive cells after 1 day of reoxygenation. However, small cells and hemocytes were dominant among these. Despite the clear effect of anoxia and reoxygenation on the number of caspase-3 cells, the number of TUNEL-positive cells per investigated volume was higher. This could indicate that the death of part of the cells was likely caused by necrosis or caspase-independent apoptosis, which can also be identified via TUNEL assays.

In conclusion, our study demonstrates that a short-term anoxic event followed by reoxygenation can activate caspase-3 and induce apoptosis in the CNS of crabs, even when the anoxic stress is mitigated by a low temperature. The transient dysregulation of neurotransmitters (NPY and dopamine) and the wave of neuronal cell death during recovery underscore the sensitivity of crustacean neural tissue to oxidative stress. These findings contribute to a deeper understanding of crustacean physiology under environmental stress and may inform strategies to improve the survivability and health of crabs during live transport.

## Data Availability

The original contributions presented in this study are included in the article. Further inquiries can be directed to the corresponding author(s).
